# Attenuation of neurovirulence of chikungunya virus by a single amino acid mutation in viral E2 envelope protein

**DOI:** 10.1186/s12929-024-00995-x

**Published:** 2024-01-17

**Authors:** Huixin Chen, Patchara Phuektes, Li Sze Yeo, Yi Hao Wong, Regina Ching Hua Lee, Bowen Yi, Xinjun Hou, Sen Liu, Yu Cai, Justin Jang Hann Chu

**Affiliations:** 1 Laboratory of Molecular RNA Virology and Antiviral Strategies, Department of Microbiology and Immunology, Yong Loo Lin School of Medicine, National University of Singapore, Singapore, Singapore; 2 Infectious Diseases Translational Research Programme, Yong Loo Lin School of Medicine, National University of Singapore, Singapore, Singapore; 3 Collaborative and Translation Unit for HFMD, Institute of Molecular and Cell Biology, Agency for Science, Technology and Research (A*STAR), Singapore, Singapore; 4 Division of Pathobiology, Faculty of Veterinary Medicine, Khon Kaen University, Khon Kaen, 40002 Thailand; 5 School of Applied Science, Republic Polytechnic, Singapore, Singapore; 6 Temasek Life Sciences Laboratory, National University of Singapore, Singapore, Singapore; 7 Department of Biological Sciences, National University of Singapore, Singapore, Singapore

**Keywords:** Chikungunya virus, Plaque size, Neurovirulence, Pathogenesis, Attenuation

## Abstract

**Background:**

Chikungunya virus (CHIKV) has reemerged as a major public health concern, causing chikungunya fever with increasing cases and neurological complications.

**Methods:**

In the present study, we investigated a low-passage human isolate of the East/ Central/South African (ECSA) lineage of CHIKV strain LK(EH)CH6708, which exhibited a mix of small and large viral plaques. The small and large plaque variants were isolated and designated as CHIKV-SP and CHIKV-BP, respectively. CHIKV-SP and CHIKV-BP were characterized in vitro and in vivo to compare their virus production and virulence. Additionally, whole viral genome analysis and reverse genetics were employed to identify genomic virulence factors.

**Results:**

CHIKV-SP demonstrated lower virus production in mammalian cells and attenuated virulence in a murine model. On the other hand, CHIKV-BP induced higher pro-inflammatory cytokine levels, compromised the integrity of the blood–brain barrier, and led to astrocyte infection in mouse brains. Furthermore, the CHIKV-SP variant had limited transmission potential in *Aedes* *albopictus* mosquitoes, likely due to restricted dissemination. Whole viral genome analysis revealed multiple genetic mutations in the CHIKV-SP variant, including a Glycine (G) to Arginine (R) mutation at position 55 in the viral E2 glycoprotein. Reverse genetics experiments confirmed that the E2-G55R mutation alone was sufficient to reduce virus production in vitro and virulence in mice.

**Conclusions:**

These findings highlight the attenuating effects of the E2-G55R mutation on CHIKV pathogenicity and neurovirulence and emphasize the importance of monitoring this mutation in natural infections.

**Supplementary Information:**

The online version contains supplementary material available at 10.1186/s12929-024-00995-x.

## Background

Chikungunya fever is an acute febrile disease caused by the Chikungunya virus (CHIKV), which can be transmitted to humans through the bite of infected *Aedes* mosquitoes [1]. CHIKV is an RNA virus that belongs to the genus *Alphavirus* of the family *Togaviridae*. It is an enveloped virus composed of a single-stranded, positive-sense RNA genome approximately about 12 kb long [2], which consists of two open reading frames (ORFs). The first ORF codes for four non-structural proteins (nsPs), nsP1 to nsP4, that are mainly responsible for the synthesis of viral RNA and processing of the polyprotein [3]. The translation of the second ORF resulted in the production of capsid, E1, E2, E3, and 6 K proteins [4]. In the host cell endoplasmic reticulum, the glycoproteins E1 and p62 (precursor of E2 and E3 proteins) combine to create heterodimers, which then trimerize into a viral spike [5]. Host cellular furin then cleaves the glycoprotein p62 into E2 and E3 during its transportation from the Golgi and early endosomes with an acidic pH to the cell membrane with a neutral pH [6]. The newly formed CHIKV particle is about 60 to 70 nm in diameter, spherical with an icosahedral (T = 4) symmetry [7].

CHIKV was first described in Tanzania in 1952–1953 [8, 9] and has since caused periodic epidemics. Over the past 60 years, numerous outbreaks of CHIKV have been documented worldwide [10]. CHIKV gained international attention following a massive outbreak on the La Réunion Island in 2005–2006, during which approximately 300,000 cases were reported. It subsequently spread to India, resulting in infection of over 1.5 million people [11]. Notably, the La Réunion outbreak was primarily transmitted by *Aedes albopictus* mosquitoes rather than *Aedes aegypti*, which is not endemic to the island [12]. Follow-up studies revealed that a solitary amino acid substitution (A226V) in the viral E1 glycoprotein was accountable for the enhanced infectivity and shortened incubation period of CHIKV in *Aedes albopictus* [13]. Additionally, neurological symptoms were initially documented in patients infected with CHIKV during this outbreak [14]. Although uncommon, neurological manifestations are the most concerning acute clinical signs due to their increased association with admissions to the intensive care unit (ICU) and death [14]. Subsequent studies have confirmed the association of CHIKV infection with neurological complications [15], and it appears that new variants of CHIKV are becoming more neurovirulent [16]. An in vivo study has provided evidence that the severity of CHIKV-induced neurological disease is dependent on the specific CHIKV strain, with infection by the ECSA lineage strain leading to more severe neurological manifestations [17]. In a prior investigation during the La Réunion outbreak of 2005–2006, out of 33 individuals who were admitted to the ICU and confirmed to have acute Chikungunya virus infection, 14 of them were found to have been diagnosed with encephalopathy [18]. In 2006, a prospective investigation of suspected Chikungunya cases in hospitalized patients from Ahmedabad and Pune, two major cities in India, with a total of 200 cases, revealed 99 cases with neurological manifestations [19]. In a more recent cohort study conducted in Brazil, which included 1410 patients admitted to the hospital neurology service, 201 of them (14%) exhibited symptoms consistent with arbovirus infection [19]. Among these 201 patients, 105 cases were associated with Chikungunya infection. The study found that Chikungunya infection was more frequently linked to central nervous system disease compared to other arbovirus infections in the cohort.

The RNA-dependent RNA polymerase of CHIKV, similar to other RNA viruses, lacks proofreading capability, resulting in exceptionally high mutation rates [20]. Consequently, viral populations consist of diverse variants, resulting in heterogeneity in plaque size [21]. Previous studies have shown that plaque morphology reflects viral replication, cell-to-cell spread, and virulence of the virus, with larger plaques often associated with increased virulence [22, 23]. Small plaque variants are commonly used as a selection criterion for attenuation due to their reduced virulence. Jaimipak et al. demonstrated the reduced virulence of a small plaque variant of a primary isolate of CHIKV in mice; however, the mechanism behind this remains unknown [24]. In this study, a low-passage human isolate of CHIKV strain LK(EH)CH6708 displayed distinct small and large viral plaques [25]. It was hypothesized that these variants would exhibit differences in replication and infectivity. The two variants, designated as CHIKV-SP and CHIKV-BP, were isolated and characterized in vitro and in vivo. CHIKV-SP exhibited lower replication in cells and mosquitoes, with the E2-55R mutation associated with its attenuation in mammals and reduced neurotropism in mice. In contrast, CHIKV-BP induced higher inflammatory responses, compromised the blood–brain barrier, and caused astrocyte infection in mouse brains.

## Materials and methods

### Cell lines and virus strain

The cell lines utilized in this study included baby hamster kidney (BHK21) cells (ATCC No. CCL-10), C6/36 cells derived from *Aedes albopictus* embryonic tissue (ATCC No. CRL-1660), and HeLa cervical cancer epithelial cells (ATCC CCL-2). C6/36 cells were cultured in Leibovitz-15 (L-15) growth medium (Sigma-Aldrich Corp., St Louis, MO, USA) supplemented with 10% heat-inactivated fetal calf serum (FCS) from Hyclone (Cramlinton, UK) at 28 °C without CO_2_. BHK21 cells were cultured in Roswell Park Memorial Institute-1640 media (RPMI-1640, Sigma-Aldrich Corp., St Louis, MO, USA) supplemented with 10% heat-inactivated FCS. HeLa cells were cultured in Dulbecco's Modified Eagle’s media (DMEM, Sigma-Aldrich Corp., St Louis, MO, USA) containing 10% heat-inactivated FCS. BHK21 and HeLa cultures were maintained at 37 °C in a humidified incubator with 5% CO_2_ throughout the study.

The CHIKV strain LK(EH)CH6708 (GenBank Accession no.: FJ513654) was obtained from a febrile CHIKV-positive patient in Singapore and kindly provided at a low-passage by the Environmental Health Institute, National Environmental Agency, Singapore.

### Viral plaque assay

Virus titration was conducted on BHK21 cells seeded at a density of 7 × 10^4^ to 8 × 10^4^ cells per well in 24-well plates (Cellstar, Greiner Bio-One, Germany) and incubated overnight at 37 °C. The virus samples were serially diluted ten-fold with RPMI-1640 containing 2% heat-inactivated FCS. Next, 100 μL of each serially diluted virus sample was added to the BHK21 cell monolayers and incubated for 1.5 h at 37 °C. The cells were then washed twice with 1 mL of phosphate-buffered saline (PBS). To overlay the cells, 1% aquacide II (Calbiochem) in RPMI containing 2% heat-inactivated FCS was added and incubated for 3 days at 37 °C with 5% CO_2_. Aquacide II is a sodium salt of carboxymethyl cellulose (CMC) with a molecular weight of 500,000, providing viscosity suitable for viral plaque assays. After the incubation period, the overlay media was removed, and the cells were fixed and stained using a solution of 10% paraformaldehyde and 1% crystal violet (Sigma-Aldrich Corp., St Louis, MO, USA) to visualize the plaques. The plates were placed on a shaker at room temperature for at least 1 day. Following staining, the solution was discarded, and the plates were washed with water before counting the plaques. The virus titres were expressed as plaque-forming units per milliliter (PFU/mL).

### Selection and purification of small and large plaque CHIKV variants

BHK cells were infected with CHIKV-LK(EH)CH6708 and incubated for 4 days to allow plaque formation. Individual small and large plaques from the terminal dilution were selected using sterile pipette tips, suspended in serum-free medium, and directly inoculated onto fresh C6/36 cells. The virus was propagated in C6/36 cells for two additional passages and then subjected to another round of plaque purification. This process was repeated until a consistent plaque morphology was observed. The resulting CHIKV variants with uniform small and large plaques were designated as CHIKV-SP and CHIKV-BP, respectively. The parental CHIKV strain, LK(EH)CH6708, was referred to as CHIKV-MP to distinguish it from the newly isolated variants.

### Replication kinetics of CHIKV-SP and CHIKV-BP in vitro

C6/36, BHK-21 and HeLa cells were seeded at a density of 1 × 10^5^ cells per well in 24-well plates, and the cell culture media was removed prior to virus inoculation. Different variants of CHIKV were inoculated at a multiplicity of infection (M.O.I) of 1 and allowed to adsorb for 1.5 h at 37 °C with 5% CO_2_. After the adsorption period, the inoculum was removed, and the monolayers were washed with PBS before the media was replaced. Supernatants of infected cells were collected at 0, 6, 12, 18, 24, 48, 72, 96, and 120 h post-infection (h.p.i.). The indicated times of infection in the data exclude the adsorption period. The titre of infectious virus particles was determined by standard viral plaque assay.

### Murine model of CHIKV infection

The animals were housed and the experiments were conducted in a pathogen-free Animal Biosafety Level 2 (ABSL-2) facility at the vivarium of the National University of Singapore. The mortality rate of CHIKV-infected 6-day-old BALB/c mice was analyzed to evaluate the virulence of different CHIKV variants. The mice were intraperitoneally inoculated with various doses of CHIKV-SP, CHIKV-BP, or CHIKV-MP, and daily monitoring was performed for a period of 14 days after infection. The severity of clinical symptoms of CHIKV infection in the mice was assessed using a mouse clinical scoring system, which evaluated criteria such as activity, breathing, movement, and change in body weight (Additional file 1: Table S1). A total score of 6 was defined as the humane endpoint. The number of survivors was recorded daily for two weeks post-infection, and any surviving mice were euthanized at the end of the experiment following humane procedures. All animal work was conducted in accordance with the guidelines provided by the Institutional Animal Care and Use Committee of the National University of Singapore, and the study was approved under IACUC Protocol No. R18-0041.

### Quantification of viral load in mouse serum and tissue

Each mouse was infected with 5 × 10^5^ PFU of CHIKV-SP or CHIKV-BP and sacrificed at 6 h and 1 to 5 days post-infection (d.p.i.). To quantify viremia, the mice were euthanized using CO_2_, and cardiac puncture was performed to collect whole blood. The blood was then centrifuged at 1000*g* for 10 min at 4 °C to obtain serum for viral plaque assay. The spleen, brain, liver, and limbs were harvested after perfusion with PBS, weighed, and homogenized in 1 mL of PBS using a Precellys^®^ 24 bead mill homogenizer (Bertin Technologies, Siège, France). Homogenization was carried out using homogenization tubes containing CK28 ceramic beads, with 3 cycles at 6500 rpm for 10 s. After homogenization, the tissue samples were centrifuged at 10,000*g* for 10 min at 4 °C to remove tissue debris. The supernatants were collected and titrated using viral plaque assays.

### Blood–brain barrier integrity

The integrity of the mouse blood–brain barrier** (**BBB) was evaluated using the Evans Blue dye permeability assay to investigate the effects of CHIKV infection. Each of 6-day-old BALB/c mice were inoculated with 5 × 10^5^ PFU of CHIKV-BP or CHIKV-SP. Mice were intraperitoneally injected with 4 μl/g of body weight of a 2% solution of Evans Blue in PBS at 2 d.p.i. Three hours later, mice were anaesthetised and perfused with PBS. The whole brain was removed and homogenized in 1000 µL of PBS. The samples were centrifuged at 15,000*g* for 30 min at 4 °C. The resulting supernatant was collected and an equal amount of 50% trichloroacetic acid was added. The samples were incubated overnight at 4 °C and then centrifuged at 15,000*g* for 30 min at 4 °C. The concentration of Evans Blue stain was measured using a spectrophotometer at a wavelength of 610 nm. The results of the Evans Blue stain quantification were presented as micrograms of Evans Blue stain per gram of tissue, indicating the permeability of the blood–brain barrier.

### Immunofluorescence assay

Brain tissues harvested from animals were fixed in a 4% paraformaldehyde solution and embedded in paraffin blocks. The blocks were then sectioned at a thickness of 4 µm following standard histological procedures. To prepare the tissue sections for staining, they underwent deparaffinization using Clearene solvent (Leica Biosystems, Germany), followed by rehydration using a series of graded ethanol to water solutions. Heat-mediated antigen retrieval was performed to enhance antigen detection.

For the detection of CHIKV, the tissue sections were incubated overnight at 4 °C with a rabbit-specific anti-dsRNA primary antibody (dilution 1:200). Subsequently, the sections were incubated with an Alexa Fluor 555 secondary antibody (Thermo Fisher Scientific, USA; dilution 1:200) for 30 min at room temperature. The anti-NeuN antibody (dilution 1:200) was employed to detect neurons, the anti-Iba1 antibody (dilution 1:200) for microglia, and the anti-GFAP antibody (dilution 1:200) for astrocytes. Each primary antibody was applied to the tissue sections for 1 h at room temperature. Following this, appropriate secondary antibodies labeled with Alexa Fluor 488 (dilution 1:200) were added and incubated for 30 min at room temperature. These secondary antibodies bind to the respective primary antibodies, enabling visualization of the specific cell types. Finally, the sections were mounted using Fluoroshield mounting media containing DAPI (Abcam, USA), and were visualized under Nikon Eclipse Ni-U Microscope.

### Mouse cytokine profiling

To evaluate the expression of a panel of cytokine genes involved in mouse immune functions, RNA was extracted from 100 μL of CHIKV-SP, CHIKV-BP, or mock-infected mouse blood (n = 6 for each group). The Qiagen RNeasy Mini Kit was used for RNA extraction according to the manufacturer’s instructions. Genomic DNA removal was carried out by incubation in GE2 buffer. Complementary DNA (cDNA) synthesis was performed using the RT^2^ First Strand Kit with 1 μg of total RNA. For PCR analysis, 550 ng of cDNA was mixed with RT^2^ SYBR Green Master mix and applied to a 96-well RT^2^ Profiler PCR array plate. The plate contained lyophilized RT^2^ qPCR primers for 84 related genes, five housekeeping genes, three reverse transcription controls, three positive PCR controls, and one mouse genomic DNA contamination control. Amplification was performed in an ABI 7500 thermocycler, and fluorescence data were collected after each cycle. Normalization was performed using the most stable genes identified by the software at the Qiagen Data Analysis Center.

### Mosquitoes oral infection and sample collection

The *Aedes albopictus* (NEA-EHI strain) was obtained from the National Environment Agency (NEA), Singapore. The colony was maintained in the insectary as described previously [26]. Mosquitoes were infected with CHIKV-BP or CHIKV-SP using a Hemotek membrane feeding system as described earlier [27]. Fresh rabbit blood was collected and separated into plasma and blood cells. The plasma was heat-inactivated, while the blood cells were washed and resuspended in the heat-inactivated plasma. The viruses were diluted in RMPI-1640 medium and mixed with the reconstituted blood to achieve a final titre of 10^7^ PFU/mL for each virus. Mosquitoes were starved overnight without water and sugar before feeding on the infected blood meal for 2 h. Non-engorged mosquitoes were removed, water and sugar were provided to the infected mosquitoes every other day. Oviposition was performed between 48 and 96 h post infection (h.p.i.). At 7 days post infection (d.p.i.), individual mosquito samples including the bodies, legs, and salivary glands were dissected in cold PBS and collected in RPMI-1640 medium. The virus titres in the supernatants were determined by plaque assay after homogenization of the samples.

### Whole genome sequencing of CHIKV

The whole genomes of CHIKV-SP and CHIKV-BP were determined through automated DNA sequencing using the BigDye™ Terminator sequencing technology. The sequencing was conducted at AIT biotech Pte Ltd, Singapore. For cDNA sequencing, viral RNA was extracted from virus-infected cell culture supernatants using the QIAamp^®^ Viral RNA Mini Kit. First-strand cDNA synthesis was performed using SuperScript™ III reverse transcriptase, and two overlapping cDNA fragments spanning the entire virus genome were amplified using Q5^®^ High-Fidelity DNA polymerase and gene-specific primers. The PCR products were then gel purified using the MinElute Gel Extraction Kit and cloned into the pSMART LC Kan vector (Lucigen). Two clones of each overlapping cDNA fragment were sequenced. Sequence analysis was conducted using Chromas™ version 2.3 and Clone Manager 5/Align Plus 4 software.

### Next-generation sequencing and data analysis

The extracted viral RNA was fragmented, and then reverse-transcribed into cDNA using the Maxima H Minus Double-Stranded cDNA synthesis kit, following the manufacturer’s protocol (Thermo Fisher Scientific, Massachusetts, USA). The synthesized cDNA was purified using the Nucleospin Gel and PCR Clean-up Kit (Macherey–Nagel, Düren, Germany) through column purification. Library preparation was conducted using the SeqCap EZ HyperCap Workflow with the KAPA Hyper Prep Kit and Single-Indexed SeqCap Adapter Kit A (Roche Diagnostics, Mannheim, Germany). For virome analyses, the paired-end FASTQ files generated from sequencing were analyzed using the cloud-based online classification tool Genome Detective, utilizing default parameters.

### Construction of full-length infectious cDNA clones of CHIKV-LK(EH)CH6708

In this study, a full-length infectious cDNA clone of LK(EH)CH6708 was constructed using two sub-genomic clones, which were then assembled into a full-length clone using unique restriction sites. Viral RNA was reverse-transcribed using SuperScript™ III reverse transcriptase (Invitrogen, Carlsbad, CA) and served as a template for PCR amplification of two viral cDNA fragments using Q5^®^ High Fidelity DNA polymerase (New England Biolabs, MA, UK). These fragments were subsequently cloned into the pSMART LC Kan vector (Lucigen). The plasmid pSMART/5´CHIKV contained nucleotides 1–5700 of LK(EH)CH6708, preceded by a T7 RNA polymerase promoter and two additional 5′ G residues to enhance transcription efficiency. On the other hand, the plasmid pSMART/3′CHIKV contained nucleotides 5578 to 11,727 of LK(EH)CH6708, followed by a 40-mer poly-A tail and unique NotI restriction sites downstream of the poly-A tail. To generate the full-length clone, a SacI-AgeI fragment was excised from the pSMART/3´CHIKV clone and cloned into the SacI-AgeI sites of pSMART/5′CHIKV. The resulting clone, named pSMART-CHIKV-6708, represented the full-length LK(EH)CH6708 genome. The clones were screened through restriction enzyme digestion and sequencing to confirm their integrity and accuracy. For cloning and amplification of all plasmid constructs, competent *E. coli* strain XL10-gold (Stratagene) was used.

### Site-directed mutagenesis

To introduce a specific mutation into the infectious clone pSMART-CHIKV-6708, site-directed mutagenesis was performed using PCR mutagenesis and the In-Fusion® HD Cloning system from Clontech. The process involved designing a primer pair that incorporated the desired nucleotide substitution. Inverse PCR was carried out using the CloneAmp™ HiFi PCR Premix (CloneTech), with a reaction mixture of 25 µL containing 5 pmol of each primer, 12.5 µL of CloneAmp HiFi PCR premix, and 100 ng of the pSMART-CHIKV-6708 plasmid. The PCR reaction consisted of 35 cycles, with denaturation at 98°C for 10 s, annealing at 55 °C for 15 s, and extension at 72 °C for 2 min. A final extension step was performed at 72 °C for 5 min. The resulting linearized PCR product was then recircularized using the In-Fusion^®^ HD Cloning system following the manufacturer’s protocol.

### Transfection and recovery of infectious clone-derived viruses

The full-length cDNA was in vitro-transcribed into RNA using the mMessage mMachine T7 transcription kit (Life Technologies), following the manufacturer’s instructions. Subsequently, the in vitro-transcribed RNA was transfected into BHK-21 cells using Lipofectamine™ 2000 (Invitrogen, Carlsbad, CA). Briefly, BHK-21 cells were seeded into 24-well tissue culture trays (Costar) at a density of 7 × 10^4^ cells per well. After incubating for 18–24 h, the cells were replaced with RPMI supplemented with 2% FBS. The lipid-nucleic acid complex, consisting of 1 μg of in vitro-transcribed RNA and 3 μL of Lipofectamine™ 2000, was added to the cells and incubated for 48 to 72 h. The clone-derived virus was collected from the cell-culture supernatant, which was clarified by centrifugation at 1000*g* for 15 min and stored at -80 ºC until needed.

### Statistical analyses

Statistical analyses were performed using GraphPad Prism 8 software. Viral titres of in vitro and mosquito studies were compared using one-way ANOVA followed by Dunnett’s test (*P < 0.05, **P < 0.01, ***P < 0.001). Survival curves were compared using log rank Mantel-Cox curve comparison. Clinical score data were compared using multiple Student’s t-tests with Holm-Sidak multiple-comparison correction. Blood–brain barrier integrity assay was analysed using Kruskal–Wallis test followed by Dunn’s multiple comparisons post-test. The Mann–Whitney U-test was conducted for cytokine expression profile study.

## Results

### The LK(EH)CH6708 isolate contains two CHIKV variants

**Fig. 1:**
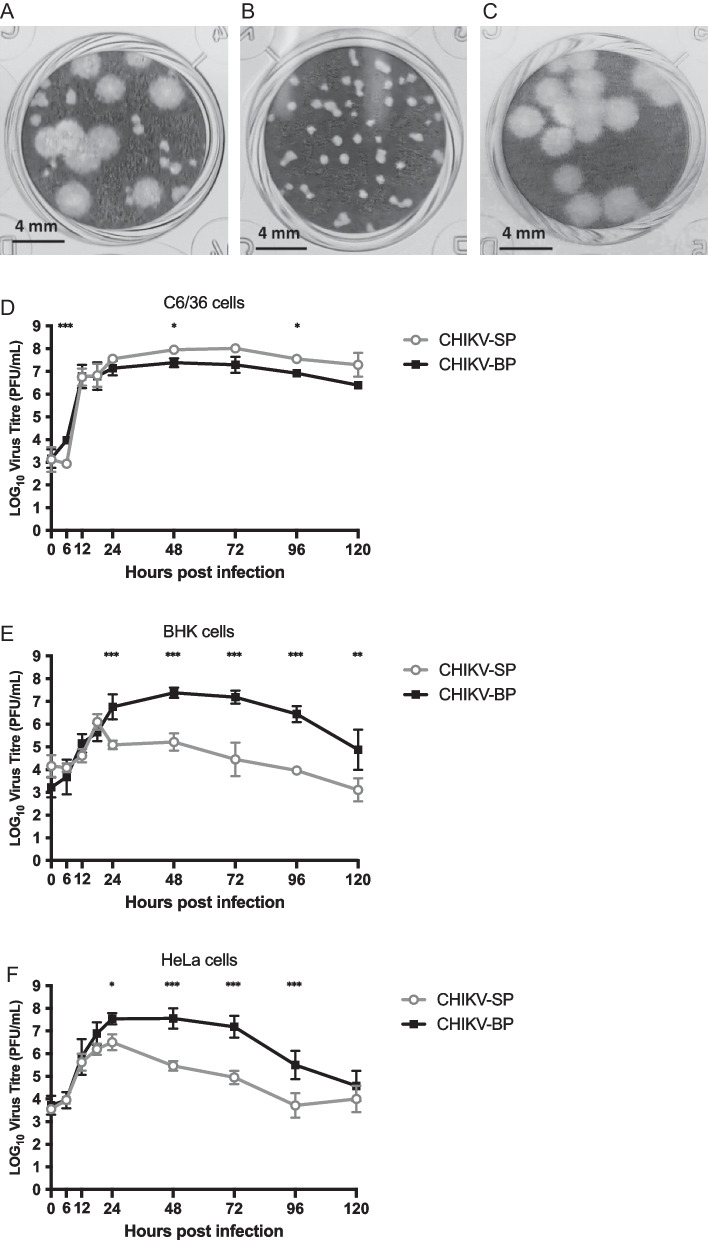
Viral plaque morphologies and replication kinetics of CHIKV variants. **A** Plaque morphologies of the LK(EH)CH6708 strain showing a mixed phenotype with both small and big plaques. Purified CHIKV-SP (**B**) and CHIKV-BP (**C**) variants displayed uniform small plaque and big plaque phenotypes, respectively. Replication kinetics of CHIKV-SP and CHIKV-BP variants were assessed in **D** C6/36 cells, **E** BHK cells, and **F** HeLa cells. Each experiment was performed in triplicates, and the results are presented as mean ± SD. Statistical analysis using one-way ANOVA followed by Dunnett’s test was conducted to compare virus titres (*P < 0.05, **P < 0.01, ***P < 0.001)

LK(EH)CH6708 isolate exhibited two distinct morphological types of plaques, namely small and big, when grown in BHK-21 cells. The average diameters of the small and big plaques were observed to be 0.33 ± 0.08 mm and 2.05 ± 0.21 mm, respectively (Fig. 1A). Following three rounds of single plaque purifications, uniform variants that formed either small or big plaques were obtained and designated as CHIKV-SP and CHIKV-BP, respectively (Fig. 1B, C).

### Growth kinetics of the CHIKV-BP and CHIKV-SP variants

To examine the growth properties of the CHIKV-SP and CHIKV-BP variants, C6/36, BHK, and HeLa cells were infected at an M.O.I. of 1. In C6/36 cells, CHIKV-SP exhibited slightly faster growth compared to CHIKV-BP starting from 18 h.p.i. The infectious titres of CHIKV-SP and CHIKV-BP in the C6/36 cell culture supernatant reached their peak at 48 h.p.i., with CHIKV-SP reaching 8.0 log_10_ PFU/mL and CHIKV-BP reaching 7.2 log_10_ PFU/mL. The difference in titres between the two variants was noticeable from 24 h.p.i. but remained relatively constant as the infection progressed (Fig. 1D). In BHK cells, CHIKV-BP exhibited significantly faster growth compared to CHIKV-SP. At 48 h.p.i., CHIKV-BP reached a maximum virus titre that was 2.3 log_10_ PFU/mL higher than CHIKV-SP (7.4 log_10_ PFU/mL compared to 5.1 log_10_ PFU/mL). From 24 h.p.i. onwards, CHIKV-BP consistently maintained an approximately 2.0 log_10_ PFU/mL higher virus titre than CHIKV-SP throughout the experiment (Fig. 1E). Similar results were observed in HeLa cells, where CHIKV-BP demonstrated faster growth than CHIKV-SP starting from 12 h.p.i. The infectious titre of CHIKV-SP in the culture supernatant reached 6.5 log_10_ PFU/mL at 24 h.p.i., which was 1.1 log_10_ PFU/mL lower than that of CHIKV-BP that yielded a maximum titre of 7.6 log_10_ PFU/mL (Fig. 1F). These findings indicate that both CHIKV-SP and CHIKV-BP variants exhibited efficient growth in C6/36, BHK, and HeLa cells. However, CHIKV-SP demonstrated a lower replicative rate in mammalian cells (BHK and HeLa cells) compared to mosquito C6/36 cells.

### Pathogenesis of CHIKV-SP and CHIKV-BP in mice

Previously, it was reported that 6-day-old BALB/c mice were susceptible to CHIKV infection [28]. In the present study, we assessed the virulence of CHIKV-SP and CHIKV-BP variants utilizing this mouse model which is well established in our laboratory. Intraperitoneal inoculation of CHIKV-SP, CHIKV-BP, or CHIKV-MP (LK(EH)CH6708) was performed in 6-day-old BALB/c mice at an infective dose of 5 × 10^6^ PFU per mouse. The survival rate, clinical scores, viremia, and viral load in various tissues were examined to assess their virulence in mice.

**Fig. 2:**
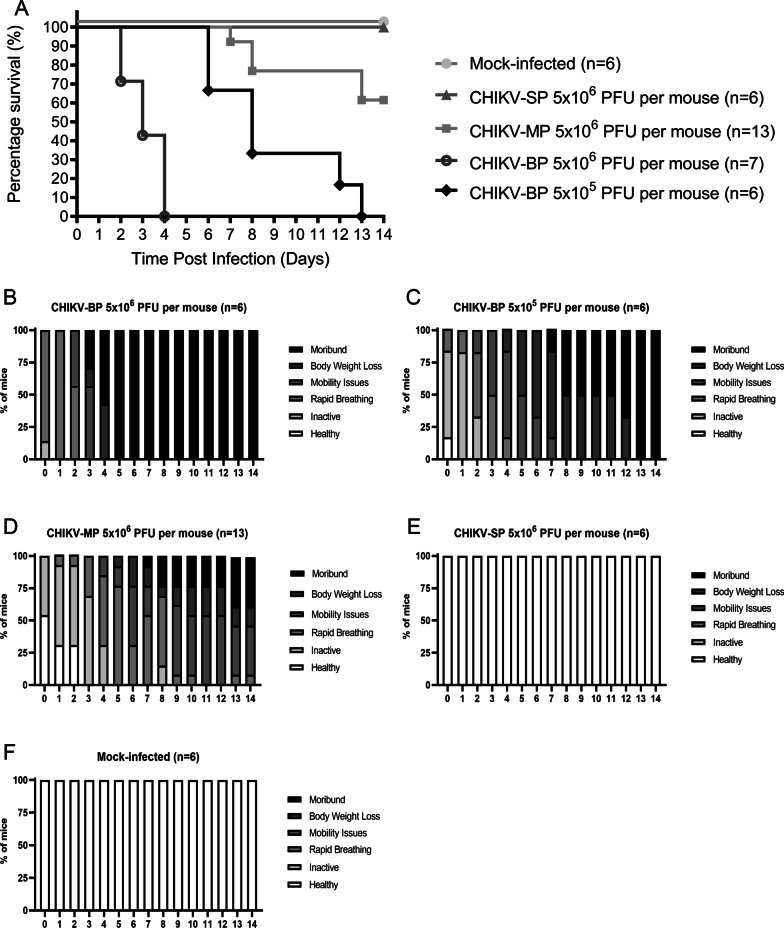

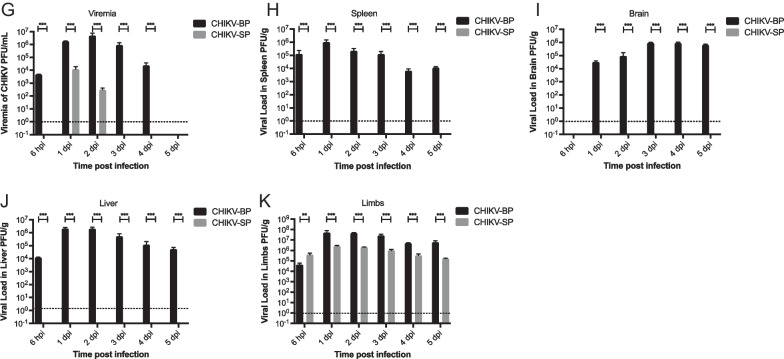
Comparison of virulence among various CHIKV variants in 6-day-old BALB/c mice model. **A** 6-day-old BALB/c mice were intraperitoneally inoculated with CHIKV-SP, CHIKV-BP, CHIKV-MP, or mock-infected. Survival was monitored daily for 14 days post-inoculation, and the data are presented as Kaplan–Meier survival curves. Statistical analysis using the Mantel-Cox test revealed significant differences (P < 0.05) between the survival curves of the CHIKV-infected groups compared to the mock-infected group. **B**–**F** Daily assessments of the mice were conducted using a mouse clinical scoring system to evaluate the severity of clinical symptoms induced by CHIKV infection (Additional file 1: Table S1). **G**–**K** 6-day-old BALB/c mice were infected with 5 × 10^5^ PFU of CHIKV-SP or CHIKV-BP. At specific time points (6 h.p.i., 1 d.p.i., 2 d.p.i., 3 d.p.i., 4 d.p.i., and 5 d.p.i.), serum, spleen, brain, liver, and limb tissues were collected from a group of 3 to 4 mice per group. The collected tissue samples were homogenized, and the supernatant was used for quantifying the amount of infectious virus particles of CHIKV-BP and CHIKV-SP using viral plaque assay. The dashed line in the figure represents the Limit of Detection (LOD) of the viral plaque assay. The mean values along with individual data points are presented to illustrate the viremia and viral load dynamics in the different tissues over the course of infection

The survival curve was generated by closely monitoring the mice daily for two weeks after inoculation, and the number of surviving mice was used to plot Kaplan–Meier survival curves (Fig. 2A). All mice (n = 6) challenged with CHIKV-SP survived at the end of the 2-week period. Mice began to succumb to CHIKV-MP infection on 7 d.p.i. with an overall survival rate of 61% (n = 13). In contrast, all mice (n = 7) infected with 5 × 10^6^ PFU of CHIKV-BP succumbed to the infection within 4 days. Furthermore, when a ten-fold lower virus titre (5 × 10^5^ PFU of CHIKV-BP per mouse) was inoculated, all mice (n = 6) succumbed to CHIKV-BP infection by 13 d.p.i. The severity of the disease, as quantified by clinical scores, is depicted in Fig. 2B–F. The clinical scores of mice infected with 5 × 10^6^ PFU of CHIKV-BP significantly increased from the onset of infection until all mice succumbed by the fifth day post-infection (Fig. 2B). Mice infected with 5 × 10^5^ PFU of CHIKV-BP also exhibited a noticeable rise in clinical scores from the onset of infection, with all mice surviving until the thirteenth day post-infection when mortality occurred (Fig. 2C). In contrast, mice infected with 5 × 10^6^ PFU of CHIKV-MP showed an increase in clinical scores from the onset of infection (Fig. 2D), but these scores were significantly lower than those of mice infected with CHIKV-BP. Correspondingly, mice infected with 5 × 10^6^ PFU of CHIKV-SP (Fig. 2E) and Mock-infected mice (Fig. 2F) maintained clinical scores at 0 throughout the 14-day period. These results indicate that CHIKV-SP exhibits a lower mortality rate than CHIKV-BP in 6-day-old BALB/c mice.

To investigate the replication and dissemination of CHIKV-BP and CHIKV-SP in mice, the infectious viral particles were quantified in various murine tissues at different time points after infection. CHIKV-BP was detected in the serum as early as 6 h.p.i., and the viral titre peaked at 2 d.p.i.. By 5 d.p.i., the virus was cleared from the bloodstream. In contrast, CHIKV-SP was only detected in the mouse serum from 1 to 2 d.p.i. (Fig. 2G). The levels of infectious CHIKV-BP virus in the spleen were measured at 3.9 log_10_ PFU/g (Fig. 2H), in the brain at 5.6 log_10_ PFU/g (Fig. 2I), in the liver at 4.6 log_10_ PFU/g (Fig. 2J), and in the limb tissues at 6.1 log_10_ PFU/g (Fig. 2K). Infectious CHIKV-BP viral particles were detected in the brain starting from 1 d.p.i., consistent with observations in other murine models [29] and human patients studies [30–32] highlighting the neurotropic nature of CHIKV and its potential to cause neurological manifestations in infected individuals. Although no infectious CHIKV-SP particles were found in the spleen, brain, or liver tissues (Fig. 2H–J), viral load was detected and persisted in the limb tissues (Fig. 2K). This is consistent with previous studies where both CHIKV-BP and CHIKV-SP exhibited the highest titres and longest persistence in the limbs [28, 33]. Based on these results, we demonstrate that CHIKV-SP is unable to replicate or disseminate systemically in the mouse body. Its replication is limited to the limb tissues. Therefore, we conclude that CHIKV-SP exhibits lower virulence than CHIKV-BP in 6-day-old BALB/c mice as it fails to cause mortality and is incapable of replicating or disseminating systemically in this mouse model.

### CHIKV-BP increase BBB permeability in vivo

**Fig. 3:**
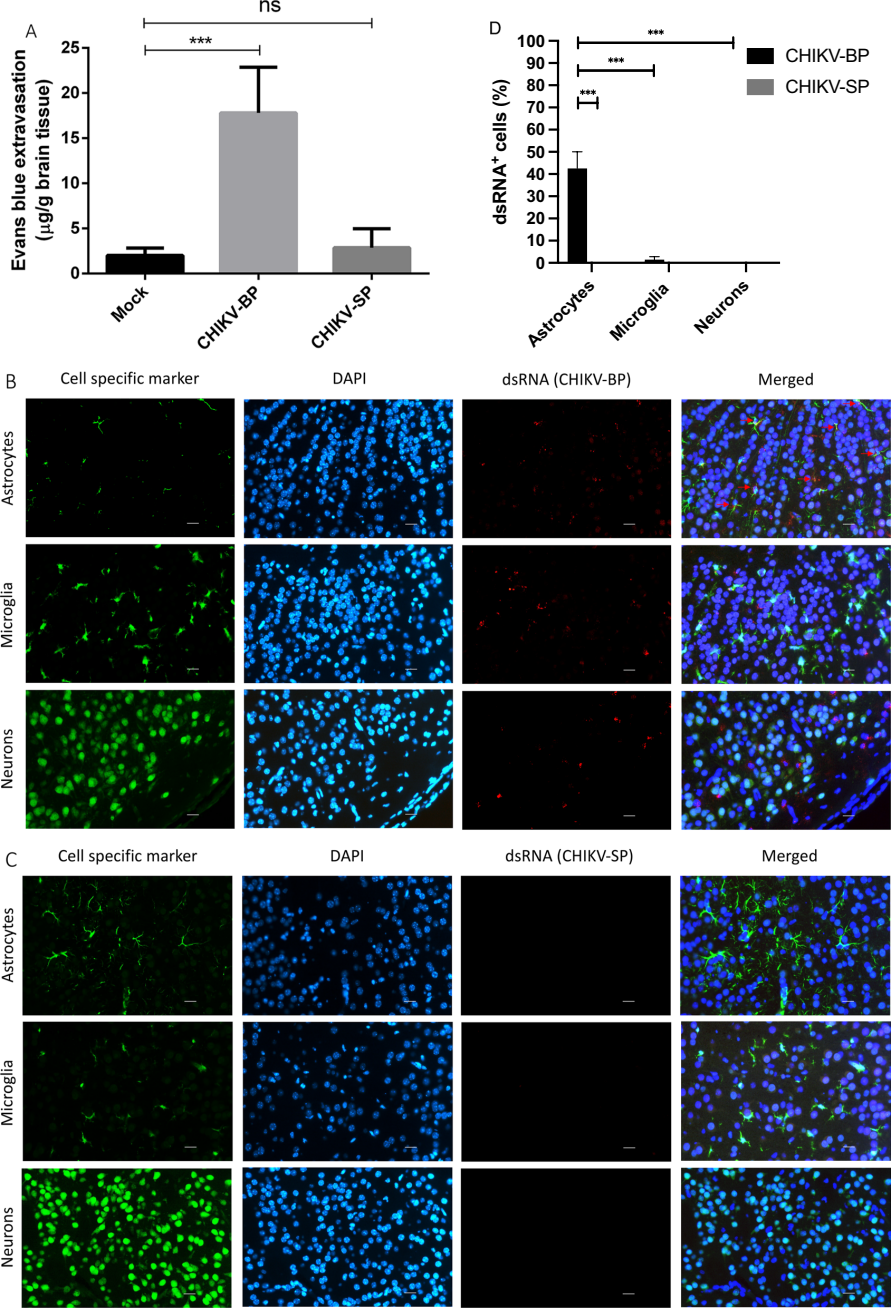
CHIKV-BP infection increases BBB permeability and selectively infect astrocytes in mouse brain. Mice were intraperitoneally injected with 4 μl/g of body weight of a 2% solution of Evans Blue in PBS at 2 d.p.i. and incubated for 3 h. **A** Quantification of Evans Blue dye incorporated into the brain was performed using spectrophotometry at 610 nm. The values represent mean ± SD (n = 6/group). Statistical analysis was conducted using Kruskal–Wallis test followed by Dunn's multiple comparisons post-test; ***p < 0.001; **p < 0.01; *p < 0.05. Brain slices from CHIKV-infected mice were subjected to double staining for dsRNA (CHIKV), the astrocyte marker GFAP, the microglia marker Iba1, and the neuron marker NeuN. **B** dsRNA-positive astrocytes were exclusively detected in CHIKV-BP-infected brains. **C** No dsRNA-positive brain cell were observed in CHIKV-SP-infected brains. Tissue sections were counterstained with DAPI. Single-channel and merged images of the same frames are presented (scale bar: 20 μm). **D** Comparative analysis of percentage (%) of dsRNA + brain cells

Evans Blue is utilized as an indicator to assess the mouse BBB integrity due to its ability to bind to albumin, a protein that normally cannot freely cross the intact BBB [34]. By binding to albumin, Evans Blue enables the detection of any leakage or increased permeability of the BBB, serving as a reliable marker for identifying potential disruptions or damage to the barrier [35] due to CHIKV infection. Comparing the CHIKV-BP infected group (n = 6) with the CHIKV-SP infected group (n = 6), a significant difference in Evans Blue extravasation into the brain tissue was observed. The mean Evans Blue extravasation (17.80 ± 2.07 μg/g of brain tissue) in the CHIKV-BP infected group was significantly higher than that in the CHIKV-SP infected group (2.87 ± 0.86 μg/g of brain tissue), indicating increased permeability of the BBB in the CHIKV-BP infection (Fig. 3A). These findings suggest that CHIKV-BP infection disrupts the integrity of the mouse BBB, leading to enhanced permeability and the leakage of Evans Blue dye into the brain parenchyma. In contrast, CHIKV-SP infection did not exhibit a similar effect on BBB integrity.

### CHIKV-BP infects astrocytes in mouse brain

To examine the cellular tropism of CHIKV in the mouse brain, specific markers (GFAP for astrocytes, Iba-1 for microglia, and NeuN for neurons) were employed to label different cell types in mouse brain tissue sections, while the dsRNA antibody was employed to stain for CHIKV. As depicted in Fig. 3B, the dsRNA signal was predominantly detected in astrocytes, indicating that CHIKV-BP primarily infects astrocytes within the mouse brain. There was no observable evidence of infection in neurons or microglial cells at 2 d.p.i. The IFA analysis did not detect any signal associated with CHIKV-SP in the mouse brain (Fig. 3C), suggesting that CHIKV-SP does not exhibit a similar tropism as CHIKV-BP. These findings were in line with the results obtained from in vivo pathogenesis study and BBB integrity assays, further supporting the tropism of CHIKV-BP towards mouse brain. Notably, astrocytes as the predominant immune cells in the brain parenchyma [36] may serve as a source of pro-inflammatory cytokines such as IL-6 and TNF-α [37], contributing to the neuroinflammatory response to CHIKV infection.

### Comparison of mouse cytokine expression profiles upon infection by CHIKV-SP and CHIKV-BP variants

**Fig. 4:**
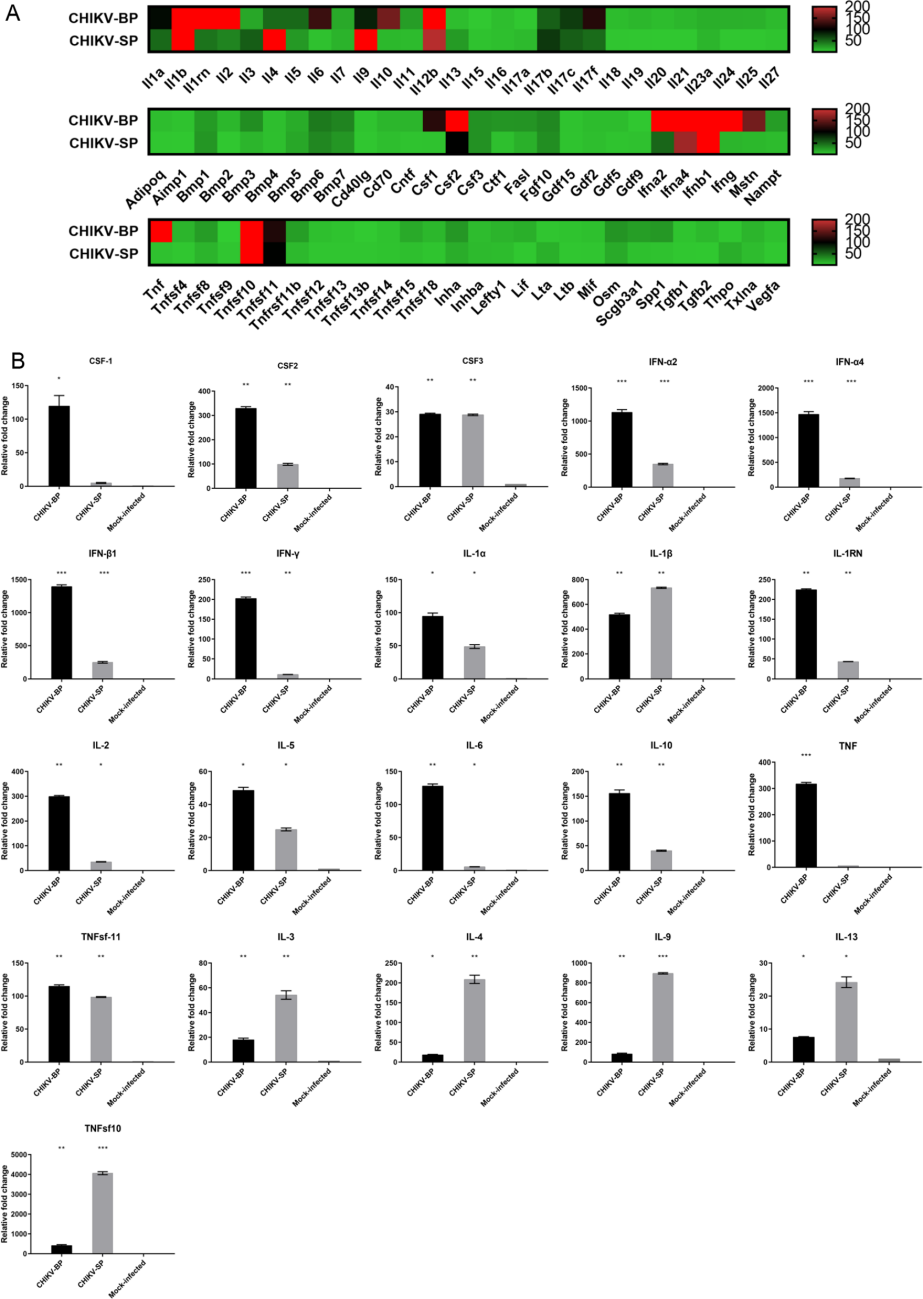
Analysis of mouse cytokine gene expression. **A** Heatmap illustrating the expression of mouse cytokine genes at 2 days post-inoculation with CHIKV-SP (n = 6) or CHIKV-BP (n = 6). High and low gene expression levels are depicted in red and green, respectively. **B** Cytokines showing significant upregulation in their expressions (≥ twofold change, P < 0.05) in CHIKV-BP or CHIKV-SP-infected mice compared to the mock-infected group (n = 6). Data are presented as mean ± SEM, and statistically significant values are denoted as *P < 0.05, **P < 0.01, ***P < 0.001 (Mann–Whitney U-test). Multiple comparison tests were performed for each group of genes and adjusted against the mock-infected group

To explore the difference in immune response to CHIKV-SP and CHIKV-BP infections in a 6-day-old mouse model, mRNA of 84 mouse cytokine genes was analyzed using the RT^2^ Profiler PCR Arrays (Qiagen, Hilden, Germany) at 2 d.p.i., comparing to mock-infected mouse serum samples. The analysis revealed a significant elevation (≥ twofold change, P < 0.05) in the expression of twenty-three genes in both CHIKV-BP and CHIKV-SP-infected mice (Fig. 4A). These genes included CSF2, CSF3, IFN-α2/α4, IFN-β1, IFN-γ, IL-1α/β, IL-1RN, IL-2, IL-5, IL-6, IL-7, IL-10, IL-27, and TNFsf-11 (Fig. 4B). Notably, CHIKV-BP infection resulted in more than 100-fold increments (p < 0.05) in the expression of CSF2, IFN-α2/α4, IFN-β1, IFN-γ, IL-1α, IL-1RN, IL-2, IL-6, IL-10, and TNFsf-11 compared to the mock-infected group (Fig. 4B) It is known that the production of pro-inflammatory cytokines including TNFα, IL-1β, IL-6 and INF-γ could increase BBB permeability. Our data suggest that the high virulence of CHIKV-BP may be attributed to the strong pro-inflammatory immune response triggered by the virus. In contrast, CHIKV-SP infection significantly upregulated (p < 0.05) IL-1β, IL-3, IL-4, IL-9, IL-13, and TNF-sf10 (Fig. 4B). The upregulation of IL-4, IL-9, and IL-13 may facilitate swift antibody production, leading to virus clearance in CHIKV-SP-infected mice.

### Replication of CHIKV-SP and CHIKV-BP in mosquitoes

**Fig. 5:**
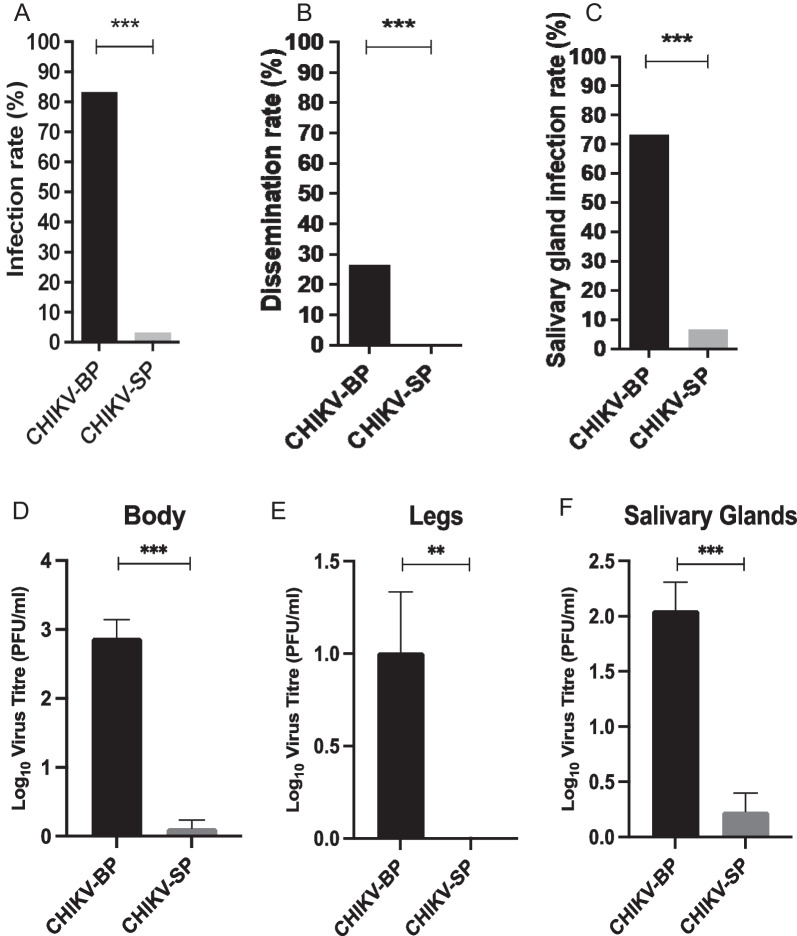
Infection rate, dissemination rate, and salivary gland infection rate of CHIKV-BP and CHIKV-SP in mosquitoes. Mosquitoes were blood-fed with CHIKV-BP or CHIKV-SP viruses at a titre of 10^7^ PFU/mL. The presence of the virus was analyzed in the bodies (as an indicator of the infection rate), legs (as an indicator of the dissemination rate), and salivary glands (indicative of the salivary gland infection rate) at 7 d.p.i. **A** The infection rate was calculated as the percentage of infected mosquitoes divided by the total number of mosquitoes examined. **B** The dissemination rate was determined by scoring the number of infected mosquitoes with infected legs out of the total number of infected mosquitoes. **C** The salivary gland infection rate was measured as the ratio of mosquito with detectable virus in salivary glands to the number of infected mosquitoes. The bars represent the cumulative data for infection, dissemination, and salivary gland infection rates from a sample size of n = 30 mosquitoes. Statistical analysis was performed using Fisher's exact test. (**D**–**F**) Demonstrates the mean ± SEM tires of CHIKV-BP and CHIKV-SP in the body, legs, and salivary glands of mosquitoes examined

In the present study, we investigated the replication capability of CHIKV-BP, and CHIKV-SP variants in *Aedes albopictus* (*Ae. albopictus*) mosquitoes. Mosquitoes were orally infected with either CHIKV-BP or CHIKV-SP using Hemotek membrane feeding systems. 7 days after the blood meal, we analyzed the presence of each CHIKV variant in the mosquito bodies, legs, and salivary glands to evaluate their infection, dissemination, and salivary gland infection rates. Our results revealed that CHIKV-BP exhibited a higher infection rate, with 83.3% of engorged mosquitoes testing positive for the virus, compared to only 3.3% for CHIKV-SP (Fig. 5A). This suggests that CHIKV-SP has reduced ability to infect *Ae. Albopictus.* Furthermore, CHIKV-BP demonstrated a 26.7% dissemination rate, as the virus was detected in the legs of infected mosquitoes, while CHIKV-SP showed no presence in the legs, indicating a defect in its ability to cross the midgut barrier to reach this site (Fig. 5B).

Finally, the salivary gland infection rate, determined by the detection of the virus in mosquito salivary glands, was 73.3% for CHIKV-BP and 6.67% for CHIKV-SP (Fig. 5C). To determine whether the decreased dissemination rate of CHIKV-SP is accompanied by lower viral titres in disseminated tissues, we measured the viral titres of CHIKV-BP and CHIKV-SP in the bodies, legs and salivary glands (Fig. 5D–F). Consistent with the dissemination rates, CHIKV-BP achieved an average titre of 2.88 log_10_ PFU/mL, 1.01 log_10_ PFU/mL, and 2.06 log_10_ PFU/mL in mosquito bodies, legs, and salivary glands at 7 d.p.i. At this time point, the CHIKV-SP titre was only 0.12 log_10_ PFU/mL and 0.23 log_10_ PFU/mL in bodies and salivary glands, respectively, while it was not detectable in legs. These findings suggest that CHIKV-SP exhibits a lower transmission potential compared to CHIKV-BP, possibly due to its reduced dissemination rate and lower infectious viral titres.

### Sequence comparison of CHIKV-SP and CHIKV-BP variants

The observed differences in plaque morphology, growth kinetics, and virulence in the mouse and mosquito models may be attributed to specific mutations present in the genomes of the CHIKV-BP and CHIKV-SP variants. To identify the genetic determinants responsible for these differences, the complete genomes of both variants were sequenced using next-generation sequencing (NGS) and automated DNA sequencing methods. Through NGS analysis, eight nucleotide differences were initially identified. However, further data analysis using LoFreq and FreeBayes revealed that only two of these nucleotide differences were confirmed (Table 1). Specifically, a non-synonymous mutation C5459T was found, resulting in an amino acid substitution from serine (S) to proline (P) at position 471 of the nsP3 protein. Additionally, a non-synonymous mutation G8677A was detected, leading to an amino acid change from glycine (G) to arginine (R) at position 55 of the E2 protein. These two nucleotide differences were subsequently validated through BigDye™ Terminator sequencing (Additional file 1: Table S2).

**Table 1:** Nucleotide differences between CHIKV-BP and CHIKV-SP based on next generation sequencing data analysis

Nucleotide Position	CHIKV-BP	CHIKV-SP	LoFreq	FreeBayes
737	G	A	3.82	Too low
932	G	A	Too low	35.59
5459	C	T	98.40	HomoAlt
5890	G	T	Too low	39.31
8149	C	T	Too low	32.72
8533	A	C	Too low	30.67
8677	G	A	97.1	HomoAlt
11,412	A	T	Too low	20.00

### E2-G55R mutation in the E2 gene is responsible for the small plaque phenotype of CHIKV in vitro

**Fig. 6:**
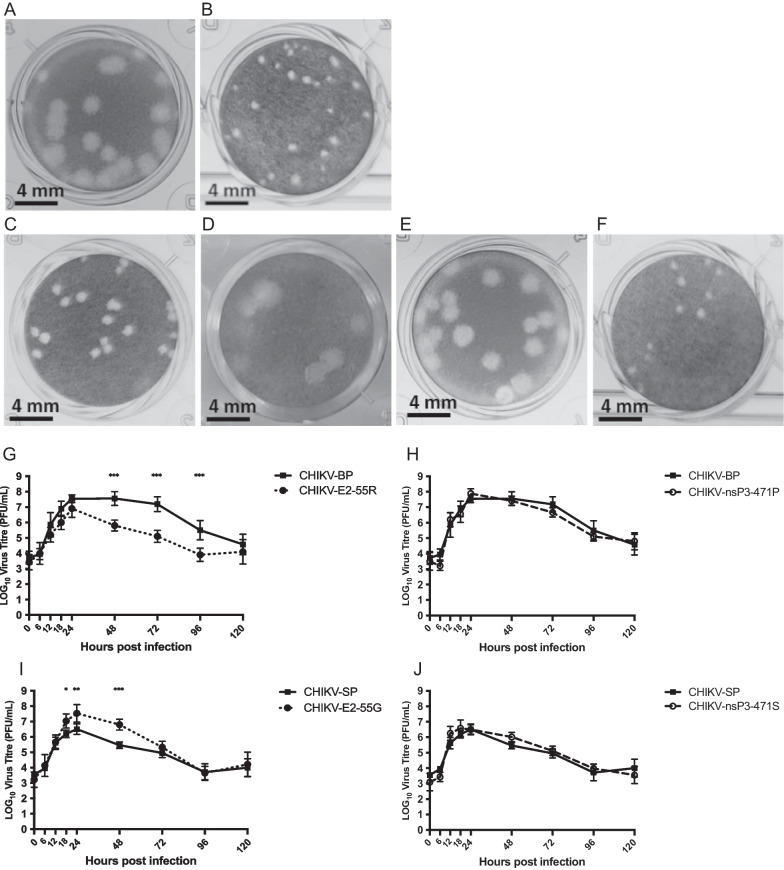

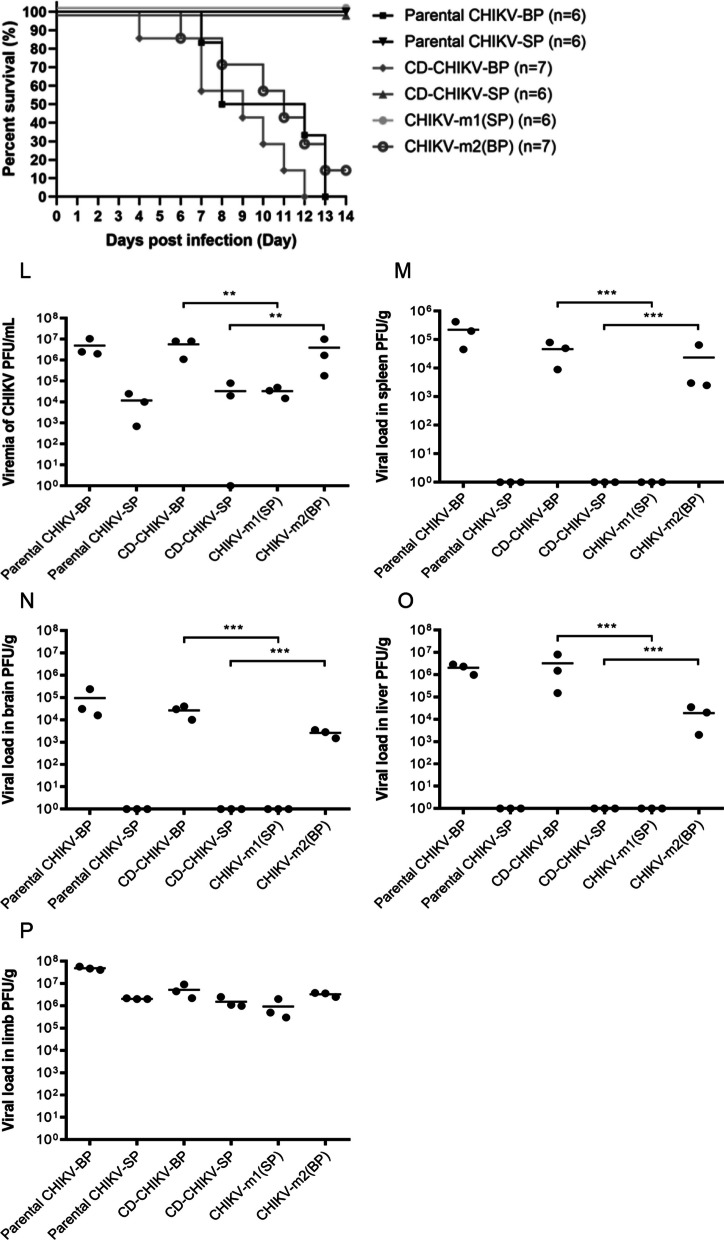
Characterization of infectious clone-derived and mutant viruses. **A** CHIKV-BP infectious clone-derived virus exhibited large plaques (2.03 ± 0.15 mm). **B** CHIKV-SP infectious clone-derived virus exhibited small plaques (0.36 ± 0.09 mm). **C** CHIKV-m1 clone-derived virus exhibited small plaques (0.38 ± 0.09 mm). **D** CHIKV-m2 clone-derived virus exhibited large plaques (2.08 ± 0.16 mm). **E** CHIKV-m3 clone-derived virus exhibited large plaques (2.11 ± 0.17 mm). **F** CHIKV-m4 clone-derived virus exhibited small plaques (0.35 ± 0.08 mm). HeLa cells were infected with each variant of CHIKV at a M.O.I. of 1. The supernatant was collected at various time points post-infection, and infectious virus particles were quantified using a standard viral plaque assay. **G** Replication kinetics of CHIKV-E2-55R (m1) and the parental variant CHIKV-BP. **H** Replication kinetics of CHIKV-nsP3-471P (m3) and the parental variant CHIKV-BP. **I** Replication kinetics of CHIKV- E2-55G (m2) and the parental variant CHIKV-SP. **J** Replication kinetics of CHIKV- nsP3-471S (m4) and the parental variant CHIKV-SP. Each experiment was performed in triplicates, and the mean ± SD are shown. Statistical analysis was conducted using one-way ANOVA followed by Dunnett’s test to compare virus titres (*P < 0.05, **P < 0.01, ***P < 0.001). **K** 6-day-old BALB/c mice were intraperitoneally inoculated with each of the clone-derived viruses (CHIKV-BP, -SP, -m1, -m2, -m3, and -m4). Survival was monitored daily for 14 d.p.i., and the data were presented as Kaplan-Meier survival curves. Statistical analysis was performed using the Kaplan-Meier method with a Mantel-Cox test. **L**–**P** At 2 d.p.i., serum, spleen, brain, liver, and limb tissues (n = 3 mice per group) were harvested. The amount of infectious virus particles in these tissues was quantified using a viral plaque assay. The mean values along with individual data points are shown

To investigate the role of nucleotide changes C5459T and G8677A in the differences observed in plaque morphology between CHIKV-SP and CHIKV-BP in vitro, reverse genetic system was utilized by generating infectious clones of these two variants. The viruses derived from these clones exhibited viability and induced cytopathic effects (CPE) in BHK cells within 24 h after transfection. Notably, the plaque phenotype of the viruses recovered from the infectious cDNA clones closely resembled that of their respective parental virus variants, as demonstrated in Fig. 6A, B.

The identified mutations were individually introduced into the CHIKV infectious clone backbone using site-directed mutagenesis and the In-Fusion^®^ HD cloning system (Clontech), as summarized in Table 2. The resulting clone-derived viruses were assessed for their plaque phenotypes, as depicted in Fig. 6C–F. Introducing the G to A mutation at position 8677, leading to a substitution of G with R at E2-55 on the CHIKV-BP backbone, yielded a clone-derived virus (CHIKV-m1) displaying a small plaque phenotype (0.38 ± 0.09 mm). Conversely, introducing the A to G mutation at position 8677, resulting in a substitution of R with G at E2-55 on the CHIKV-SP backbone, generated a clone-derived virus (CHIKV-m2) exhibiting a large plaque phenotype (2.08 ± 0.16 mm). In contrast, no significant change in plaque phenotype was observed when the C nucleotide at position 5459 was mutated to T (CHIKV-m3) or vice versa (CHIKV-m4).

**Table 2:** Mutations incorporated into infectious clones by site-directed mutagenesis

Clone	Backbone	Nucleotide 5459	Nucleotide 8677	Amino acid nsP3-471	Amino acid E2-55
CHIKV-BP		···C···	···G···	Serine (Ser)	Glycine (Gly)
CHIKV-SP		···T···	···A···	Proline (Pro)	Arginine (Arg)
CHIKV-m1	CHIKV-BP	···C···	···A···	Serine (Ser)	Arginine (Arg)
CHIKV-m2	CHIKV-SP	···T···	···G···	Proline (Pro)	Glycine (Gly)
CHIKV-m3	CHIKV-BP	···T···	···G···	Proline (Pro)	Glycine (Gly)
CHIKV-m4	CHIKV-SP	···C···	···A···	Serine (Ser)	Arginine (Arg)

### E2-G55R mutation reduces the virus production of CHIKV in vitro

After confirming that the plaque phenotypes of the clone-derived viruses resembled those of their parental variants, the viral replication kinetics were investigated in vitro to assess the impact of the two mutations. HeLa cells were infected with the vector-derived CHIKV-m1, CHIKV-m2, CHIKV-m3, CHIKV-m4, CHIKV-BP, and CHIKV-SP at a M.O.I. of 1, and the viral replication kinetics were monitored for five consecutive days. As depicted in Fig. 6G, CHIKV-m1 carrying the E2-G55R mutation exhibited a significantly lower viral peak at 6.9 log_10_ PFU/mL compared to its parental variant, CHIKV-BP, which peaked at 7.6 log_10_ PFU/mL. In contrast, CHIKV-m3 harboring the nsP3-S471P mutation showed no significant difference in growth kinetics compared to its parental variant, CHIKV-BP (Fig. 6H). Figure 6I demonstrates that the E2-G55R mutation in CHIKV-m2 resulted in a significantly higher viral peak at 7.5 log_10_ PFU/mL compared to its parental variant, CHIKV-SP, which peaked at 6.5 log_10_ PFU/mL. Conversely, CHIKV-m4 carrying the nsP3-P471S mutation did not exhibit a significant difference in growth kinetics compared to its parental variant, CHIKV-SP (Fig. 6J).

### E2-G55R mutation reduces the virulence of CHIKV in vivo

To determine if the E2-G55R mutation reduces the virulence of CHIKV in mice, clone-derived (CD)-CHIKV-SP, CD-CHIKV-BP, CHIKV-m1, and CHIKV-m2 were intraperitoneally inoculated into 6-day-old BALB/c mice at an infective dose of 5 × 10^5^ PFU per mouse. The mice were observed for a period of 14 days, and the number of surviving mice was used to construct Kaplan–Meier survival curves. The results showed that mice infected with CD-CHIKV-BP started to succumb to the infection from 4 days post-infection (d.p.i.), and all mice (n = 7) had died by 12 d.p.i., as depicted in Fig. 6K. Similarly, mice infected with CHIKV-m2 began to succumb to the infection from 6 d.p.i., resulting in an overall survival rate of 14% (n = 7). In contrast, all mice challenged with CD-CHIKV-SP (n = 6) or CHIKV-m1 (n = 6) survived at the end of the 14-day period. These survival study findings indicate that both CD-CHIKV-SP and CHIKV-m1, carrying the E2-G55R mutation, exhibited reduced virulence as no mice succumbed to the infection.

To investigate the replication and dissemination of clone-derived CHIKV-m1 and CHIKV-m2 in mice, the infectious viral particles were quantified in various murine tissues at 2 d.p.i.. In mice infected with CHIKV-m1, the viremia titre was measured to be 3.5 log_10_ PFU/mL, which was not significantly different from the viremia levels observed in mice infected with the parental CHIKV-SP (3.1 log_10_ PFU/mL) or CD-CHIKV-SP (3.5 log_10_ PFU/mL) (Fig. 6L). On the other hand, mice infected with CHIKV-m2 exhibited a viremia of 6.6 log_10_ PFU/mL at 2 d.p.i. This viremia level was comparable to that of the parental CHIKV-BP (6.7 log_10_ PFU/mL) and CD-CHIKV-BP (6.8 log_10_ PFU/mL), but significantly higher than the viremia level observed in CHIKV-m1-infected mice. Similar trends were observed in the viral load analysis of the spleen (Fig. 6M), brain (Fig. 6N), liver (Fig. 6O), and limb tissues (Fig. 6P). Notably, no infectious CHIKV-m1 viral particles were detected in the mouse spleen, brain, or liver, and the viral load in mouse limb tissues was lower compared to CHIKV-m2-infected mice. These findings indicate that CHIKV-m1 is unable to replicate or disseminate systemically in mice, with limited replication observed in the limb tissues. Therefore, our results suggest that the E2-G55R mutation, carried by the CHIKV-SP variant, is responsible for its lower virus production in vitro and low virulence in the established 6-day-old BALB/c mouse model.

## Discussion

In this study, we characterized two variants of the LK(EH)CH6708 isolate of CHIKV, namely CHIKV-SP and CHIKV-BP, which exhibited distinct morphological differences in plaque formation. The CHIKV-SP variant formed small plaques, while the CHIKV-BP variant formed large plaques. The significance of having two variants of CHIKV coexisting in nature is unclear. It has been reported that the size of a viral plaque is often associated with the pathogenicity of the virus [22, 23]. Thoka and colleagues (2018) identified three mutations, nsP2-L618, nsP3-R117, and E2-K187, are responsible for the big plaque phenotype of CHIKV isolated from patient in Thailand [38]. Sukkaew and colleagues (2018) demonstrated that the large-plaque, but not small-plaque, patient isolated viruses are potent stimulators of inflammatory cytokines in vitro [39]. Recently, Jaimipak and colleagues (2018) showed that the small plaque size variant of CHIKV primary isolate has reduced virulence in mice [24]. In the present study, these variants were isolated through single plaque purifications and further amplified in C6/36 cells. CHIKV may exist as a mixed population of viral variants genetically linked through mutations, which is a result of its high mutation rates caused by the lack of proofreading activity in RNA-dependent RNA polymerases [20]. Quasispecies theory proposes that natural selection by the environment might impose selective pressures on the different viral variants, leading to their competition and observed distribution that is based on their replicative fitness [20]. The viral quasispecies could benefit under a unique selective environment where it could alternate its replication between mosquito vectors and mammalian hosts. To investigate the growth kinetics of CHIKV-SP and CHIKV-BP, we infected C6/36, BHK, and HeLa cells and monitored viral titres at different time points. CHIKV-SP demonstrated slightly faster growth in C6/36 cells compared to CHIKV-BP, while CHIKV-BP exhibited significantly faster growth in BHK and HeLa cells. These results indicate that both variants can efficiently replicate in different cell types, but CHIKV-SP has a lower replicative rate in mammalian cells compared to mosquito cells. Further studies are needed to investigate the roles of the quasispecies synergistic effect in CHIKV replication. Intriguingly, our study revealed a noteworthy phenomenon where CHIKV-SP exhibited a mild increase in replication within C6/36 mosquito cells, but paradoxically displayed impaired replication within the mosquito vector when compared to CHIKV-BP. This discrepancy suggests an intricate interaction between viral strains and their respective hosts, emphasizing the complex adaptations that occur in distinct environments. Notably, the distinct immune responses in mosquitoes compared to a cell line, along with potential tissue-specific restrictions or barriers in live mosquitoes, may contribute to these variations. Further investigations into the underlying mechanisms driving these differences are warranted. Such insights could have broader implications in the realm of vector control and our understanding of viral transmission dynamics, thereby contributing to more effective strategies for managing CHIKV and related arboviral diseases.

Several CHIKV outbreaks have been caused by viruses with novel mutations in both the old and new worlds. The causes of the outbreak, potential threats and burden to public health, and the effectiveness of prevention and control are all areas of concerns [40]. The 2005–2006 La Réunion Island epidemic which infected nearly 300,000 individuals, was related to a strain of CHIKV that had a mutation in the viral envelope glycoprotein gene (E1-A226V) [41]. Lamballerie and colleagues (2008) demonstrated that this mutation dramatically increases the ability of CHIKV to infect *Ae. albopictus* mosquitoes and no additional adaptive mutations are required to gain intermolecular compatibility [42]. Follow-up studies discovered E1-A226V has a number of impacts on CHIKV physiology, including dependency for cholesterol and lower pH, as well as mosquito infectivity [43]. In another study, Gorchakov and colleagues (2012) reported that the attenuated CHIKV strain 181/25 is produced by substituting two amino acid residues (E2-T12I and E2-G82R) in the E2 envelope protein [44]. Follow-up studies discovered that E2 residue 82 is a key driver of GAG utilization, and that it is mainly responsible for the attenuation of strain 181/25 [45]. In a subsequent study, although the detailed mechanisms remain unclear, introduce the E1-K211E or E2-V264A mutation into a CHIKV background of 226A but not 226 V resulted in enhanced infectivity and transmission in *Aedes aegypti* [46]. Recently, the E2-K200R mutation was reported to be predominantly responsible for the increased viral pathogenicity and spread in mice [47]. However, the mechanisms through which this mutation affects viral spread and pathogenicity are still unknown. These discoveries suggested that key mutations on the viral genome could have significant impacts on the epidemiology and expansion of the virus. In the present study, we have identified residue 55 on the E2 glycoprotein as a genomic virulence factor of CHIKV which is responsible for small plaque phenotype and reduced virulence in mice. The E2 glycoprotein of CHIKV is made up of 423 amino acids, which are organized into three domains resembling immunoglobulin-like structures: A, B, and C. The receptor binding site is in domain A, while domain B is positioned at the outermost part of each spike and shields the fusion loop on Domain II of E1. Domain C is situated nearest to the viral membrane. A ribbon links domain B to domains A and C. The E2-55 amino acid is found within a cleft, surrounded by other sections of Domain A, but it is oriented outward from the inner core of the spike, positioned next to the β ribbon connector. In a previous study, mouse Mxra8, a cell adhesion molecule, was identified as a receptor for multiple arthritogenic alphaviruses, including CHIKV, Ross River virus (RRV), Mayaro virus (MAYV), and O'nyong-nyong virus (ONNV) [48]. In a recent structure-guided mutation study, the authors identified three amino acids as direct interface residues (W64, D71, and I121 in the A domain of E2) [49, 50], while G55 is not directly involved in binding to Mxra8. Similar to the findings from a recent study by Hawman and colleagues (2017), high CHIKV titres were observed to persist in mouse limb as skeletal muscles and joints are the predominant targets for CHIKV infection and this is in agreement with viral tropism in human muscles and joints [47]. It is intriguing that no correlation was observed between the persistent CHIKV-SP viral load in mouse limb tissues and the infection outcome of our mouse model. CHIKV-SP being less potent in triggering inflammatory response and causing tissue damage could be a plausible explanation. Studies in human patients showed that CHIKV replication in skeletal muscle is associated with severe outcomes, but the clinical outcome of muscle tissue due to CHIKV infection has not been fully understood.

Studies have demonstrated that CHIKV has the ability to replicate and spread to brain tissue in mice, leading to neuropathological alterations and neuroinflammatory responses. A recent study reported the detection of CHIKV in the mouse brain as early as 2 days after infection [51]. This infection leads to increased expression of various genes associated with viral infection, including IL-6 and TLR3 [52]. Astrocytes, which can express these genes during viral neuroinflammation, have been shown to be susceptible to CHIKV infection [36]. Similarly, in human infections, CHIKV can traverse the blood–brain barrier, enter the central nervous system, replicate, and elicit inflammatory responses within the brain. In a recent investigation, researchers conducted a comparative analysis of the cytokine profile in patients who experienced neurological complications and those who did not after being infected with CHIKV [53]. The level of 4 cytokines (TNF-α, IFN-α, IL-6, and monokine induced by IFN-γ) were found to be significantly higher in CHIKV patients with neurological diseases. Serum IL-1β and IL-6 were also identified as biomarkers of CHIKV disease severity in a recent study of CHIKV infected patients [54]. However, the role of these cytokines in disease pathogenesis is still unclear. In the present study, we explored the mouse cytokine profiles following infection with CHIKV-SP or CHIKV-BP. There was a significant elevation in the pro-inflammatory cytokines, including IL-1β, IL-6, IFN-α/γ and TNF-α in CHIKV-BP-infected mice. The pathogenesis of viral infections is generally aided by an increase in pro-inflammatory cytokine response. A systemic cytokine storm may occur as a result of severe inflammation, which can lead to more severe pathological damage [55]. On the other hand, IL-4, IL-9, IL-13, IL-17 were significantly elevated in CHIKV-SP-infected mice. IL-4 is important for mouse IgE isotype switching and development of TH2 cells. IL-9 is mainly generated by CD4 + cells which have been shown to be involved in infectious diseases, such as clearance of respiratory syncytial virus [56]. Production of IL-4 and IL-13 in the early phase of CHIKV infection was proposed as markers to predict persistent joint pain in patients [57]. Early production of IL-13 together with IL-4 was reported to prevent immune-mediated arthritis caused by CHIKV infection [58]. In CHIKV infections, IL-17 has been linked to swelling joint [58]. Elevation of IL-4, IL-9 and IL-13 in CHIKV-SP-infected mice could result in swift antibody production leading to virus clearance.

Several cytokines have been implicated in increasing the permeability of the mouse BBB. These cytokines include TNF-α, IL-1β, IL-6, IL-8, and IL-17 [59, 60]. These pro-inflammatory cytokines can disrupt the tight junctions between endothelial cells of the BBB, leading to increased permeability and allowing immune cells and pathogens, to cross into the brain tissue during neuroinflammatory processes.

These findings emphasize the differential cellular preferences of CHIKV variants and raise intriguing questions about the role of astrocytes in CHIKV pathogenesis. The specific targeting of astrocytes by CHIKV-BP may contribute to distinct mechanisms of viral replication and pathogenicity within the central nervous system. Further investigations are warranted to elucidate the underlying molecular interactions between CHIKV-BP and astrocytes, which may provide valuable insights into the pathogenesis and potential therapeutic strategies for Chikungunya virus infection.

Given that adult immunocompetent mice are not susceptible to CHIKV infection, six-day-old mice were employed in the present study. It is important to acknowledge that our use of 6-day-old mice as a model system may present certain limitations in extrapolating our findings to human populations susceptible to CHIKV infection and neurological disease. The developmental stage and immune responses of neonatal mice differ significantly from those of adult humans, making it crucial to exercise caution when drawing direct parallels. The unique neurodevelopmental characteristics, immune system, and susceptibility to viral infections in neonates could impact the manifestation and severity of CHIKV-associated neurological outcomes in a manner distinct from the human population. Therefore, while our study provides valuable insights into the pathogenesis of CHIKV, further research employing models or age groups more representative of the human condition is warranted to enhance the translational relevance of our findings and to address the potential variations that may arise in different age groups.

## Conclusion

In this study, two distinct variants of the CHIKV virus, CHIKV-SP and CHIKV-BP, were characterized based on their plaque morphologies. CHIKV-SP formed small plaques, while CHIKV-BP formed large plaques. The presence of such variants in nature underscores the importance of considering plaque size in assessing viral pathogenicity.

To evaluate the growth kinetics of these variants, we infected different cell lines and monitored viral titers. CHIKV-SP showed slightly faster growth in mosquito cells (C6/36) compared to CHIKV-BP, while CHIKV-BP exhibited significantly faster growth in mammalian cells (BHK and HeLa). These results suggest that both variants can efficiently replicate in diverse cell types, but CHIKV-SP has a reduced replicative rate in mammalian cells compared to mosquito cells.

The virulence of CHIKV-SP and CHIKV-BP was assessed in a mouse model. CHIKV-SP displayed lower mortality in 6-day-old BALB/c mice compared to CHIKV-BP. Additionally, viral replication and dissemination in mouse tissues were examined. CHIKV-BP was detected in the serum as early as 6 h post-infection (h.p.i.), with the virus reaching peak titers at 2 days post-infection (d.p.i.). The virus was also found in various tissues, including the spleen, brain, liver, and limb tissues. In contrast, CHIKV-SP was only detected in the serum at 1–2 d.p.i. and exhibited limited replication, primarily in limb tissues, indicating its inability to systemically replicate or disseminate in mice.

The integrity of the blood–brain barrier (BBB) was assessed using Evans Blue dye, revealing that CHIKV-BP infection increased BBB permeability, while CHIKV-SP did not exhibit a similar effect. Further investigation into cellular tropism demonstrated that CHIKV-BP primarily infected astrocytes in the mouse brain, potentially contributing to neuroinflammatory responses.

A comparative analysis of cytokine expression in infected mice revealed distinct profiles for CHIKV-SP and CHIKV-BP. CHIKV-BP induced higher levels of pro-inflammatory cytokines, including IL-1β, IL-6, IFN-α/γ, and TNF-α, which may contribute to increased BBB permeability and virulence. Conversely, CHIKV-SP-infected mice showed elevated levels of IL-4, IL-9, IL-13, and IL-17, possibly facilitating antibody production and virus clearance.

Additionally, the genetic basis for these differences was explored. Sequencing revealed two key mutations in CHIKV-SP compared to CHIKV-BP: a non-synonymous mutation (C5459T) in the nsP3 protein and another non-synonymous mutation (G8677A) in the E2 protein. Introduction of the E2-G55R mutation into CHIKV-BP resulted in a small plaque phenotype and reduced virulence in mice, highlighting its role as a genomic virulence factor.

In conclusion, our findings elucidate the differential characteristics of CHIKV variants, providing insights into their replication, virulence, and genetic determinants. Understanding these differences contributes to our knowledge of CHIKV pathogenesis and may inform future therapeutic strategies for Chikungunya virus infections.

### Supplementary Information

**Additional file 1: Table S1.** Mouse clinical scoring system in the CHIKV Balb/C mouse model. **Table S2.** Nucleotide and amino acid differences between CHIKVBP and CHIKV-SP based on BigDye™ Terminator sequencing data analysis.

## Data Availability

All data supporting the findings of this study are available within the paper and its Supplementary Mouse clinical scoring system in the CHIKV Balb/C mouse model are provided in Additional file 1: Table S1, Nucleotide and amino acid differences between CHIKV-BP and CHIKV-SP based on BigDye™ Terminator sequencing data analysis are provided in Additional file 1: Table S1.

## Abbreviations

ABSL-2: Animal biosafety level 2
*Ae.*: *Aedes*
BBB: Blood–brain barrier
BHK: Baby hamster kidney
cDNA: Complementary DNA
CHIKV: Chikungunya virus
CMC: Carboxymethyl cellulose
CPE: Cytopathic effects
DMEM: Dulbecco’s Modified Eagle’s media
d.p.i.: Days post-infection
ECSA: East/Central/South African
FCS: Fetal calf serum
Fig.: Figure
G: Glycine
h.p.i.: Hours post-infection
ICU: Intensive care unit
IFN: Interferon
IL: Interleukin
L-15: Leibovitz-15
MAYV: Mayaro virus
min: Minutes
M.O.I.: Multiplicity of infection
NEA: National Environment Agency
NGS: Next-generation sequencing
nsP: Non-structural protein
ONNV: O’ Nyong Nyong virus
ORF: Open reading frame
P: Proline
PBS: Phosphate-buffered saline
PCR: Polymerase chain reaction
PFU: Plaque-forming units
R: Arginine
RNA: Ribonucleic acid
RPMI: Roswell Park Memorial Institute
RRV: Ross River virus
S: Serine
TLR: Toll-like receptor
TNF: Tumor necrosis factor
