# Supplementary material for: Attenuation of neurovirulence of chikungunya virus by a single amino acid mutation in viral E2 envelope protein
Source: J Biomed Sci. 2024 Jan 17;31:8. doi: 10.1186/s12929-024-00995-x (PMC10792792; doi:10.1186/s12929-024-00995-x)
Supplement: Supplementary file 1 — Additional file 1: Table S1. Mouse clinical scoring system in the CHIKV Balb/C mouse model. Table S2. Nucleotide and amino acid differences between CHIKVBP and CHIKV-SP based on BigDye™ Terminator sequencing data analysis. [file 12929_2024_995_MOESM1_ESM.pdf]

Table S1. Mouse clinical scoring system in the CHIKV Balb/C mouse model.

	**0**	**1**	**2**	**3**
**Activity**	Normal	Isolated, lethargy, abnormal posture	Huddled, inactive or overactive	Moribund or seizuring
**Breathing**	Normal	Rapid, shallow	Rapid, abdominal	Laboured
**Movement**	Normal	Slight incoordination	Single limb dragging/ paralysis	Staggering, paralysis, multiple limbs dragging
**Body Weight**	Normal (Gaining weight 0.51g/day)	Stopped or markedly reduced growth (5% loss over 24h)	Weight loss>15% or up to 10% in 24h	Weight loss>20% or >10% over 24h

Table S2. Nucleotide and amino acid differences between CHIKVBP and CHIKV-SP based on BigDye™ Terminator sequencing data analysis.

	Position	CHIKV-BP	CHIKV-SP
Nucleotide	5459	C	T
	8677	G	A
Amino acid residue	nsP3-471	Serine (Ser)	Proline (Pro)
	E2-55	Glycine (Gly)	Arginine (Arg)
